# Essential Components of an Electronic Patient-Reported Symptom Monitoring and Management System: A Randomized Clinical Trial

**DOI:** 10.1001/jamanetworkopen.2024.33153

**Published:** 2024-09-13

**Authors:** Kathi Mooney, Mary Gullatte, Eli Iacob, Natalya Alekhina, Bridget Nicholson, Elizabeth A. Sloss, Jennifer Lloyd, Ann Marie Moraitis, Gary Donaldson

**Affiliations:** 1 College of Nursing, University of Utah, Salt Lake City; 2 Huntsman Cancer Institute, University of Utah, Salt Lake City; 3 Emory University, Atlanta, Georgia (Retired); 4 College of Nursing, The University of Rhode Island, Kingston; 5 School of Medicine, University of Utah, Salt Lake City

## Abstract

**Importance:**

Multicomponent electronic patient-reported outcome cancer symptom management systems reduce symptom burden. Whether all components contribute to symptom reduction is unknown.

**Objective:**

To deconstruct intervention components of the Symptom Care at Home (SCH) system, a digital symptom monitoring and management intervention that has demonstrated efficacy, to determine which component or combination of components results in the lowest symptom burden.

**Design, Setting, and Participants:**

This randomized clinical trial included participants who were older than 18 years, had been diagnosed with cancer, had a life expectancy of 3 months or greater, were beginning a chemotherapy course planned for at least 3 cycles, spoke English, and had daily access and ability to use a telephone. Eligible participants were identified from the Huntsman Cancer Institute, University of Utah (Salt Lake City), and from Emory University Winship Cancer Institute, including Grady Memorial Hospital (Atlanta, Georgia), from August 7, 2017, to January 17, 2020. Patients receiving concurrent radiation therapy were excluded. Dates of analysis were from February 1, 2020, to December 22, 2023.

**Interventions:**

Participants reported symptoms daily during a course of chemotherapy and received automated self-management coaching with an activity tracker without (group 1) and with (group 2) visualization, nurse practitioner (NP) follow-up for moderate-to-severe symptoms without (group 3) and with (group 4) decision support, or the complete SCH intervention (group 5).

**Main Outcomes and Measures:**

The primary outcome, symptom burden, was assessed as the summed severity of 11 chemotherapy-related symptoms rated on a scale of 1 to 10 (with higher scores indicating greater severity), if present.

**Results:**

The 757 participants (mean [SD] age, 59.2 [12.9] years) from 2 cancer centers were primarily female (61.2%). The most common cancer diagnoses were breast (132 [17.4%]), lung (107 [14.1%]), and colorectal (99 [13.1%]) cancers; 369 patients (48.7%) had metastatic disease. The complete SCH intervention including automated self-management coaching and NP follow-up with decision support (group 5) was superior in reducing symptom burden to either of the self-management coaching groups, as shown by the mean group differences in area under the curve (group 1, 1.86 [95% CI, 1.30-2.41] and group 2, 2.38 [95% CI, 1.84-2.92]; both *P* < .001), and to either of the NP follow-up groups (group 3, 0.57 [95% CI, 0.03-1.11]; *P* =.04; and group 4, 0.66 [95% CI, 0.14-1.19]; *P* = .014). Additionally, NP follow-up was superior to self-management coaching (group 1 vs group 3, 1.29 [95% CI, 0.72-1.86]; group 1 vs group 4, 1.20 [95% 12 CI, 0.64-1.76]; group 2 vs group 3, 1.81 [95% CI, 1.25-2.37]; and group 2 vs group 4, 1.72 [95% CI, 1.17-2.26]; all *P* < .001), but there was no difference between the 2 self-management coaching groups (−0.52 [95% CI, −1.09 to 0.05]; *P* = .07) or between the 2 NP groups (−0.10 [95% CI, −0.65 to 0.46]; *P* = .74).

**Conclusions and Relevance:**

In this randomized clinical trial of adult participants undergoing chemotherapy treatment for cancer, the complete intervention, rather than any individual component of the SCH system, achieved the greatest symptom burden reduction. These findings suggest that a multicomponent digital approach to cancer symptom management may offer optimal symptom burden reduction.

**Trial Registration:**

ClinicalTrials.gov Identifier: NCT02779725

## Key Points

**Question:**

In a multicomponent digital symptom monitoring and management intervention previously shown to reduce symptom burden, what components are necessary to achieve the highest symptom burden reduction?

**Findings:**

This randomized clinical trial of 757 participants from 2 US cancer centers found that the complete multicomponent Symptom Care at Home intervention, including both automated self-management coaching and clinician follow-up for moderate and severe symptoms, achieved the greatest symptom burden reduction compared with individual components alone.

**Meaning:**

The findings of this study suggest that multicomponent digital approaches to cancer symptom management offer optimal reduction in symptom burden and efficient and improved symptom control during chemotherapy treatment for cancer.

## Teaser

This randomized clinical trial evaluates whether components or a combination of components of a patient-reported symptom tracking system contribute to the lowest symptom burden for patients with cancer undergoing chemotherapy.

## Introduction

Patients undergoing cancer treatment experience multiple co-occurring symptoms and adverse effects that are commonly reported at moderate-to-high severity.^1,2,3,4^ Systematic remote monitoring and treatment of patient-reported symptoms through digital health tools may reduce symptom burden and unplanned health care utilization.^5,6,7,8,9,10,11,12,13^

Current standard care to treat symptoms includes multiple anticipatory pharmacological prescriptions and written information on self-management with an instruction to call the oncology team if additional guidance is needed.^14,15^ However, patients rarely do so. A previous study by investigators from our team found that only 5% of the time, when symptoms were moderate to severe, did patients call their oncology team.^8^ In the presence of multiple high-intensity symptoms, patients may experience negative effects without further intervention or seek emergency department care when symptoms become intolerable.^16^

Symptoms reported once per treatment cycle at clinic visits do not capture the full symptom trajectory.^11,17^ Electronic patient-reported outcome (ePRO) symptom platforms use a variety of internet and telephone technologies to monitor and manage symptoms, facilitating more frequent monitoring. Digital health tools can also deliver self-management coaching using algorithms tailored to the specific symptom patterns reported, pairing just-in-time suggestions to the patient’s specific experience. Additionally, alert notifications can be sent to clinicians about poorly controlled symptoms that require further intervention.^5,8,9,10^ Symptom control may be enhanced by clinician use of symptom decision support systems, but limited studies have examined their value.^18,19,20,21^

Comprehensive, multicomponent interventions to improve symptom outcomes can address myriad factors that interfere with effective symptom care, including timeliness, frequency of assessment, early intervention, intensification of care before symptoms escalate, and use of best evidence for treatment.^22,23^ However, when a multicomponent intervention, tested as a bundle, is found efficacious, we do not know whether all components contribute to the improved symptom outcomes.^24,25^

Investigators from our team previously reported on the development and testing of Symptom Care at Home (SCH), an automated digital tool using interactive voice-response technology that includes symptom monitoring, automated self-management coaching based on symptoms reported, and alert notifications sent to nurse practitioners (NPs) for telephone follow-up using a decision support system.^8,26^ In a 2-group randomized clinical trial, the SCH system significantly reduced symptom severity, lowering symptom burden for SCH participants by 3.59 severity points on average.^8^ However, the investigators did not determine what components of the intervention were necessary to achieve these benefits. To address this question, we conducted a 5-group randomized clinical trial to deconstruct the SCH intervention. Our primary aim was to determine the effect of each SCH subcomponent and the complete SCH intervention on the primary outcome, overall symptom burden (severity of 11 symptoms combined), to determine which components were necessary for optimal symptom burden reduction. We hypothesized that the complete SCH intervention would provide the greatest symptom burden reduction among the groups.

## Methods

### Design

This randomized clinical trial was guided by the Chronic Care Model, an evidence-based, patient-centered model of care that emphasizes care delivery systems that are proactive with planned, systematic interventions rather than those that are acute and reactive.^27^ We used a prospective, randomized, longitudinal design to deconstruct and identify the relative contribution of the intervention elements to achieve reduced symptom burden across 5 groups (Table 1). The study was approved by the University of Utah and Emory University Institutional Review Boards, as well as by Grady Memorial Hospital. Eligible patients were approached by study staff at the visit prior to their chemotherapy infusion. The study was explained, and those agreeing provided written informed consent. The study followed the Consolidated Standards of Reporting Trials (CONSORT) reporting guideline.

**Table 1.** Treatment Groups

Study group	Treatment condition	Rationale
Group 1	Automated self-management coaching	Just-in-time self-care coaching based on reported symptoms
Group 2	Automated self-management coaching; activity tracking	Just-in-time self-care coaching based on reported symptoms with activity tracking to increase physical activity engagement and provide feedback on sleep
Group 3	NP follow-up; NP best practices (in lieu of decision support)	NP follow-up when symptoms exceed alert thresholds using the NP’s best symptom treatment practices
Group 4	NP follow-up; SCH decision support for NPs	NP follow-up when symptoms exceed alert thresholds using the integrated SCH decision support system
Group 5 (complete SCH)	Automated self-management coaching; NP follow-up; SCH decision support for NPs	Complete SCH intervention with all intervention components

Abbreviations: NP, nurse practitioner; SCH, Symptom Care at Home.

The SCH system collects daily patient-reported presence and severity of 11 common chemotherapy symptoms: fatigue, trouble sleeping, nausea or vomiting, pain, numbness or tingling, feeling blue or down, feeling nervous or anxious, distress over appearance, diarrhea, sore mouth, and trouble thinking or concentrating. The intervention provides automated symptom self-management coaching paired with the reported symptoms and severity level. Alert reports about poorly controlled symptoms (moderate to severe) are monitored by study-based NPs who provide follow-up care using a symptom decision support guideline–based system. The following groups were included: group 1: automated self-management coaching with an activity tracker blinded to the participant; group 2: automated self-management coaching with an activity tracker visible to the participant to motivate activity and sleep self-management; group 3: NP follow-up when symptoms exceeded alert thresholds using the NP’s best treatment judgment; group 4: NP follow-up when symptoms exceeded alert thresholds using the SCH decision support system; and group 5: the complete SCH intervention with all components used in the prior efficacy study^8^ (self-management coaching and NP follow-up using the decision support system and self-management coaching without an activity tracker, as it was not included in the efficacy study) (Table 1) (trial protocol in Supplement 1).

### Participant Eligibility and Recruitment Sites

Patient eligibility included those over 18 years, with a cancer diagnosis, with a life expectancy of 3 months or greater, beginning a chemotherapy course planned for at least 3 cycles, who were English speaking, and with daily access and the ability to use a telephone. Patients were excluded if they were receiving concurrent radiation therapy since they would have daily contact with oncology clinicians. Participants were compensated by 2 payments of $75 each. We recruited participants from the medical oncology practices at the Huntsman Cancer Institute, University of Utah (Salt Lake City), and Emory University Winship Cancer Institute, including Grady Memorial Hospital (Atlanta, Georgia) with accrual between August 7, 2017, and January 17, 2020.

### Treatment Conditions

All groups reported symptoms daily through the SCH interactive voice-response digital tool on the presence and severity of the 11 symptoms. Interactive voice-response systems do not require the internet or a smartphone as digital data are transmitted over telephone lines, thus offering greater access than web- or application-based tools. We chose not to provide a usual care condition or a group that solely reported symptoms daily because our team’s previous research had shown that monitoring alone is not efficacious; it is necessary but not an active ingredient of the intervention effect.^8^ From the previous study, we knew that the SCH intervention significantly reduced symptom burden; therefore, we did not have clinical equipoise for randomization to usual care, as it would knowingly disadvantage participants.^8^ All groups received usual care in addition to the assigned intervention component and were reminded at the end of each reporting session to call their oncology team if they had concerning symptoms.

#### Self-Management Coaching

The automated, algorithm-based self-management coaching (groups 1, 2, and 5) was delivered during the monitoring call. Coaching was based on national evidence-based guidelines (eg, the National Comprehensive Cancer Network, the Oncology Nursing Society).^8^ Developed and validated by a national panel of symptom experts, algorithms enable personalized coaching content based on reported severity level (mild, moderate, or severe) and contextual responses (eg, vomiting frequency). Audio-recorded messages also have decision rules that include the number of times the message can be repeated. Messages add about 30 seconds to reporting length.^26,28^

#### Self-Management Coaching and Activity Tracker 

Group 1 received self-management coaching and an activity tracker with data blinded to the participant to control for any effect of wearing a device alone as compared with group 2. An activity tracker was provided to group 2 to evaluate its use and potential to enhance self-management by providing patient feedback on activity and sleep. While our team did not use an activity tracker in the efficacy study,^8^ we saw the opportunity to enhance our self-management coaching by inclusion of a commercially available activity tracker for this study.

#### NP Follow-Up for Moderate-to-Severe Symptoms 

Alert notifications were automatically and immediately generated from the patient’s daily report based on preset moderate-to-severe thresholds. The NPs for group 3 reviewed alert data on the SCH dashboard and followed up with the participant using their best professional judgment.

#### NP Follow-Up for Moderate-to-Severe Symptoms With a Decision Support System

 Groups 4 and 5 used the same SCH alert dashboard as group 3 but had different NPs who also used the SCH decision support system. The decision support system provided additional assessment data to access from the electronic health record (eg, blood counts) and areas to assess during the follow-up; it also included guideline-based pharmacological and nonpharmacological strategies as well as potential referrals.^26,28^ The complete SCH (group 5) was similar to group 4, but participants also received automated self-management coaching.

### Treatment Fidelity

As several components of the SCH system are automated, treatment fidelity was maintained because it was consistently delivered on set algorithms. The collection of the patient-reported data and delivery of self-management coaching had no variation. The NPs’ adherence to delivery of the decision support system–guided follow-up care was tracked in groups 4 and 5 to assure intervention fidelity. When adherence was below 90%, based on auditing intervention voice recordings, the decision support system intervention standards were reviewed with the NPs.

### Measures

We collected sociodemographic data from patient self-reports and disease- and treatment-related variables from the electronic health records. Race and ethnicity were self-reported and were assessed because of documented disparities in symptom management. Race and ethnicity categories included Black, Hispanic, non-Hispanic, White, and other (including American Indian or Alaska Native, Asian, Native Hawaiian or Other Pacific Islander, and multiple races). The primary outcome was overall symptom burden, defined as the cumulative severity of the 11 daily reported symptoms. The SCH system records the presence of the 11 symptoms during the past 24 hours and, if present, the severity on a scale of 1 to 10 (with higher scores indicating greater severity). We used single-item measures of symptoms to keep response burden to a minimum, given that a daily cadence has been shown to be the optimal frequency to capture symptom fluctuations.^11,17,29^ Single-item symptom measures have been found to be reliable, valid, and usable for both patients and clinicans.^26,29,30,31^

### Procedures

Participants were randomly assigned to a treatment condition. Random assignment, generated through REDCap software, was determined in blocks of 10 (at equal probability) with either 2 per arm or 3 per block, stratified by sex and independently at each of the 2 recruitment sites. Following randomization, staff collected baseline data, oriented the participant to group assignment, and trained the participant in using the SCH system. Participants remained in the study until treatment protocol completion or 6 months, whichever came first.

### Statistical Analysis

#### Sample Size and Power and Descriptive Statistics

A priori analysis indicated that targeted recruitment group samples of n = 150 would yield a power of 86% to reject a null hypothesis of end point equality at a significance threshold of *P* < .05, 2-tailed, against a small-to-moderate effect size of 0.35. Basic descriptive statistics were used for demographic and clinical descriptors. Reporting compliance was based on the proportion of reports received compared with the number expected. Summed daily symptom severity item scores for the 11 symptoms comprised the symptom burden–dependent variable, while the randomization group was the independent variable. We analyzed data using SPSS Statistics, version 27 (IBM Inc) and SAS/STAT, version 9.4 (SAS Institute Inc). Dates of analysis were from February 1, 2020, to December 22, 2023.

#### Unconditional Inferential Analysis

The area under the smoothed daily trajectories estimated the total amount of symptom burden experienced over the trial in each condition. We conducted all formal hypothesis tests using this end point (Supplement 1). Net differences between treatments were obtained by subtracting the respective areas. As there was no control condition, the magnitudes of these differences were of primary substantive interest.

Chance baseline differences were adjusted formally through the analysis model; site was included as a covariate in the conditional inferential analyses. The constrained longitudinal data analysis model^30,31^ assumed a single prerandomization population, with differences emerging only after initiation of the randomized treatment. All daily data points were incorporated in the intent-to-treat analysis, no symptom burden data were imputed, and no data were deleted. Under the population model, the parameter estimates maximized the likelihood of the vector of observed data points.

## Results

### Demographic and Clinical Characteristics

A total of 884 participants (71.1%) consented of the 1244 patients found eligible to participate, of whom 757 reported symptoms at least once and received treatment (mean [SD] age, 59.2 [12.9] years; 463 female [61.2%]; 293 male [38.7%]). Participants were primarily non-Hispanic (722 [95.4%]) and married or with a partner (474 [62.6%]). Among the participants, 240 (31.7%) were Black, 29 (3.8%) were Hispanic, 488 (64.5%) were White, and 29 (3.8%) were of other race and ethnicity. The most common cancer diagnoses were breast (132 [17.4%]), lung (107 [14.1%]), and colorectal (99 [13.1%]) cancers, and nearly half had metastatic disease (369 [48.7%]). Participants were randomized to a component group (eFigure in Supplement 2). There were no differences among groups regarding demographic and clinical characteristics (Table 2).

**Table 2.** Demographic and Clinical Characteristics of Participants Who Received Treatment by Randomization Group^a^

Characteristics	Participant group					Total treatment received (N = 757)
	Group 1 (n = 143)	Group 2 (n = 144)	Group 3 (n = 148)	Group 4 (n = 155)	Group 5 (n = 167)	
Age, y						
Mean (SD)	59.4 (13.5)	58.5 (12.7)	58.7 (13.9)	59.8 (128)	59.4 (12.3)	59.2 (12.9)
Median, range	61 (29-88)	60 (23-84)	62 (19-81)	60 (23-81)	60 (26-89)	60 (19-89)
Sex						
Female	89 (62.2)	87 (60.4)	89 (60.1)	96 (61.9)	102 (61.1)	463 (61.2)
Male	54 (37.8)	56 (38.9)	59 (39.9)	59 (38.1)	65 (38.9)	293 (38.7)
Missing	0	1 (0.7)	0	0	0	1 (0.1)
Race						
Black	48 (33.6)	40 (27.8)	45 (30.4)	48 (31.0)	59 (35.3)	240 (31.7)
White	88 (61.5)	99 (68.8)	94 (63.5)	106 (68.4)	101 (60.5)	488 (64.5)
Other^b^	7 (4.9)	5 (3.5)	9 (6.1)	1 (0.6)	7 (4.2)	29 (3.8)
Ethnicity						
Hispanic	10 (7.0)	2 (1.4)	8 (5.4)	3 (1.9)	6 (3.6)	29 (3.8)
Non-Hispanic	133 (93.0)	140 (97.2)	138 (93.2)	150 (96.8)	161 (96.4)	722 (95.4)
Missing	0	2 (1.4)	2 (1.4)	2 (1.3)	0	6 (0.8)
Marital status						
Married or with a partner	95 (66.4)	89 (61.8)	96 (64.9)	103 (66.5)	91 (54.5)	474 (62.6)
Widowed	6 (4.2)	8 (5.6)	8 (5.4)	7 (4.5)	9 (5.4)	38 (5.0)
Divorced or separated	22 (15.4)	23 (16.0)	21 (14.2)	23 (14.8)	33 (19.8)	122 (16.1)
Never married or single	20 (14.0)	21 (14.6)	23 (15.5)	21 (13.5)	34 (20.4)	119 (15.7)
Missing	0	3 (2.1)	0	1 (0.6)	0	4 (0.5)
Educational level						
Less than high school degree	5 (3.5)	4 (2.8)	10 (6.8)	7 (4.5)	8 (4.8)	34 (4.5)
High school or equivalent (eg, GED)	27 (18.9)	27 (18.8)	28 (18.9)	40 (25.8)	36 (21.6)	158 (20.9)
Some college or associate degree	55 (38.5)	57 (39.6)	45 (30.4)	47 (30.3)	54 (32.3)	258 (34.1)
Bachelor’s degree	30 (21.0)	36 (25.0)	43 (29.1)	39 (25.2)	45 (26.9)	193 (25.5)
Graduate degree	26 (18.2)	18 (12.5)	21 (14.2)	22 (14.2)	24 (14.4)	111 (14.7)
Missing	0	2 (1.4)	1 (0.7)	0	0	3 (0.4)
Yearly income, $						
≤24 999	16 (11.2)	16 (11.1)	19 (12.8)	28 (18.1)	31 (18.6)	110 (14.5)
25 000-49 999	30 (21.0)	38 (26.0)	46 (31.1)	47 (30.3)	42 (25.1)	203 (26.8)
50 000-74 999	30 (21.0)	23 (16.0)	28 (18.9)	19 (12.3)	38 (22.8)	138 (18.2)
75 000-99 999	14 (9.8)	18 (13.0)	12 (8.1)	10 (6.5)	8 (4.8)	62 (8.2)
100 000-124 999	5 (3.5)	7 (4.9)	7 (4.7)	12 (7.7)	6 (3.6)	37 (4.9)
≥125 000	13 (9.1)	11 (7.6)	12 (8.1)	12 (7.7)	16 (9.6)	64 (8.5)
Not known or preferred not to answer	30 (21.0)	29 (20.1)	22 (14.9)	23 (14.8)	20 (12.0)	124 (16.4)
Missing	5 (3.5)	2 (1.4)	2 (1.4)	4 (2.6)	6 (3.6)	19 (2.5)
Cancer diagnosis						
Breast	26 (18.2)	26 (18.0)	26 (17.6)	20 (12.9)	34 (20.4)	132 (17.4)
Lung	22 (15.4)	18 (13.0)	20 (13.5)	21 (13.5)	26 (15.6)	107 (14.1)
Colorectal	18 (12.6)	22 (15.0)	17 (11.5)	17 (11.0)	25 (15.0)	99 (13.1)
Ovarian	9 (6.3)	11 (7.6)	14 (9.5)	9 (5.8)	20 (12.0)	63 (8.3)
Pancreatic	11 (7.7)	10 (6.9)	10 (6.8)	18 (11.6)	13 (7.8)	62 (8.2)
Other	57 (39.9)	57 (40.0)	61 (41.2)	70 (45.2)	49 (29.3)	294 (38.8)
Metastatic cancer						
Without	63 (44.1)	67 (46.5)	74 (50.0)	63 (40.6)	78 (46.7)	345 (45.6)
With	72 (50.3)	68 (47.2)	67 (45.3)	83 (53.5)	79 (47.3)	369 (48.7)
Missing	8 (5.6)	9 (6.3)	7 (4.7)	9 (5.8)	10 (6.0)	43 (5.7)

Abbreviation: GED, General Educational Development.

^a^ Data are presented as No. (%) unless otherwise indicated.

^b^ Includes American Indian or Alaska Native, Asian, Native Hawaiian or Other Pacific Islander, and multiple races.

Of the 884 participants who consented, 757 (85.6%) reported symptoms at least once, and 127 (14.4%) never began the intervention (18 became ineligible; 109 never participated due to changing their mind about participating once home, disease burden, or unstated reasons). There was no difference by group assignment of nonparticipants, but they were more likely to be Black, with a lower educational attainment, or unmarried.

### Symptom Reports and Compliance

There were a total of 37 743 symptom reports with a mean (SD) of 47.9 (39.4) reports per participant (median 41.0 [range, 1.0-180.0]). Overall mean (SD) report compliance of expected reports was 69.6% (25.6%) with a median of 75.6% (range, 3.9%-100.0%) (Table 3). Of the 757 participants who received treatment, 195 (25.8%) discontinued study participation before their chemotherapy protocol was completed. The mean (SD) length of the study was 72.6 (42.2) days (participants remained in the study until their treatment protocol was complete or terminated or had lasted for 6 months, whichever came first). There were no significant differences in reporting compliance or mean length of study among groups.

**Table 3.** Symptom Reports and Compliance by Randomization Group

Symptom reports and compliance	Participant group					Total
	Group 1	Group 2	Group 3	Group 4	Group 5	
No. of participants who called	143	144	148	155	167	757
Study participation, days on study						
Mean (SD)	68.4 (41.8)	74.9 (39.6)	72.8 (40.7)	72.5 (43.1)	74.3 (45.6)	72.6 (42.2)
Median (range)	63.0 (2.0-188.0)	70.5 (3.0-194.0)	66.5 (4.0-212.0)	63.0 (4.0-183.0)	64.0 (6.0-190.0)	65.0 (2.0-212.0)
Total calls in system, No.	6739	7395	7488	7739	8382	37 743
Total calls made, mean (SD)	45.2 (37.5)	48.9 (38.2)	49.1 (38.7)	48.1 (40.0)	48.1 (42.4)	47.9 (39.4)
Total calls made, median (range)	39.0 (1.0-169.0)	48.0 (1.0-171.0)	41.0 (1.0-179.0)	40.0 (1.0-180.0)	39.0 (1.0-173.0)	41.0 (1.0-180.0)
Call compliance, %						
Mean (SD)	71.0 (25.0)	69.8 (25.0)	71.9 (24.5)	70.0 (26.6)	66.0 (26.4)	69.6 (25.6)
Median (range)	77.0 (10.0-100.0)	74.6 (14.5-100.0)	78.8 (11.8-100.0)	78.3 (5.3-100.0)	69.9 (3.9-100.0)	75.6 (3.9-100.0)

### Symptom Prevalence

Of the 757 participants, most (637 [84.1%]) reported at least 1 moderate-to-severe–level symptom (≥4 on a scale of 1 to 10). Fatigue, pain, and trouble sleeping were the most common moderate-to-severe symptoms reported.

### Overall Symptom Burden by Group

The primary outcome was symptom burden over time (Table 4 and Figure). The mean symptom burden values equivalent to the areas under the curve were 6.42 (95% CI, 5.73-7.10) for group 1; 6.94 (95% CI, 6.26-7.62), group 2; 5.13 (95% CI, 4.45-5.80), group 3; 5.22 (95% CI, 4.55-5.89), group 4; and 4.56 (95% CI, 3.90-5.22), group 5, with lower values indicating lower daily mean symptom burden over the entire course of treatment. We found the complete SCH intervention (group 5) had a significantly lower mean symptom burden than any other group (group 1, 1.86 [95% CI, 1.30-2.41]; *P* < .001; group 2, 2.38 [95% CI, 1.84-2.92]; *P* < .001; group 3, 0.57 [95% CI, 0.03-1.11]; *P* = .04; and group 4, 0.66 [95% CI, 0.14-1.19]; *P* = .01). Neither of the 2 automated self-management coaching groups (−0.52 [95% CI, −1.09 to 0.05]; *P* = .07) nor the 2 NP groups (−0.10 [95% CI, −0.65 to 0.46]; *P* = .74) differed significantly. The NP groups were statistically superior to both self-management coaching groups (group 1 vs group 3, 1.29 [95% CI, 0.72-1.86]; group 1 vs group 4, 1.20 [95% CI, 0.64-1.76]; group 2 vs group 3, 1.81 [95% CI, 1.25-2.37]; and group 2 vs group 4, 1.72 [95% CI, 1.17-2.26]; all *P* < .001).

**Table 4.** Mixed-Effects Model Results for Symptom Burden Across Time Between Groups

Group	Outcome across time	*P* value
**Study group, mean symptom burden (95% CI)** ^a^		
Group 1	6.42 (5.73 to 7.10)	<.001
Group 2	6.94 (6.26 to 7.62)	<.001
Group 3	5.13 (4.45 to 5.80)	<.001
Group 4	5.22 (4.55 to 5.89)	<.001
Group 5	4.56 (3.90 to 5.22)	<.001
**Group comparison, mean difference (95% CI)** ^b^		
Group 1 vs group 2	−0.52 (−1.09 to 0.05)	.07
Group 1 vs group 3	1.29 (0.72 to 1.86)	<.001
Group 1 vs group 4	1.20 (0.64 to 1.76)	<.001
Group 1 vs group 5	1.86 (1.30 to 2.41)	<.001
Group 2 vs group 3	1.81 (1.25 to 2.37)	<.001
Group 2 vs group 4	1.72 (1.17 to 2.26)	<.001
Group 2 vs group 5	2.38 (1.84 to 2.92)	<.001
Group 3 vs group 4	−0.10 (−0.65 to 0.46)	.74
Group 3 vs group 5	0.57 (0.03 to 1.11)	.04
Group 4 vs group 5	0.66 (0.14 to 1.19)	.01

Abbreviation: AUC, area under the curve.

^a^ Daily symptom burden was computed as the sum of 11 chemotherapy-related symptoms rated on a scale of 1 to 10 (with higher scores indicating greater severity). Estimates and 95% CIs are the mean daily scores over time as measured by AUC.

^b^ Mean group differences in AUC were measured as first group minus second group.

**Figure.**
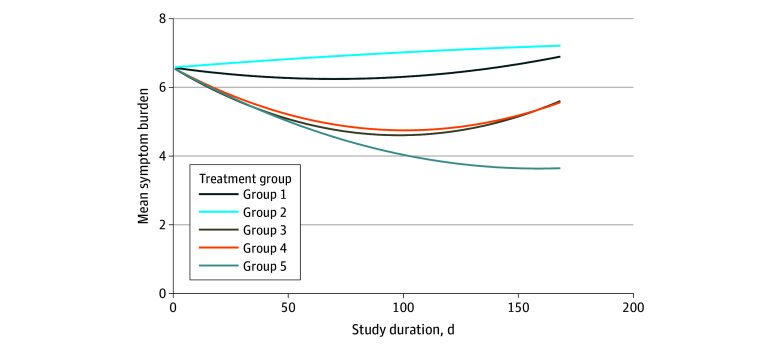
Symptom Burden Outcome by Day and Treatment Group Symptom burden was assessed as the mean summed severity of 11 chemotherapy-related symptoms rated on a scale of 1 to 10 (with higher scores indicating greater severity). Group 1: automated self-management coaching and activity tracker without participant visualization; group 2: automated self-management coaching and activity tracker with participant visualization; group 3: nurse practitioner follow-up without the Symptom Care at Home (SCH) decision support; group 4: nurse practitioner follow-up with the SCH decision support; and group 5: a combination of automated self-management coaching and nurse practitioner follow-up with SCH decision support.

## Discussion

Multicomponent interventions facilitate targeting several aspects of a clinical problem.^22,23^ When these interventions are found to be efficacious, it is important to know if all components contribute to patient benefit prior to their implementation in routine care. The complete SCH targets both patient self-management strategies and clinician notification with decision support. In this randomized clinical trial, we found that these components combined resulted in the greatest symptom burden reduction when compared with the 4 subcomponent groups.

Patients in the complete SCH group experienced more than 1 half-point decrease in symptom burden than did the next-closest NP groups over the entire duration. Given recent evidence that shows even mild symptom burden can lead to patient bother and treatment discontinuation,^32^ this consistent reduction has clinical significance for patients living with the daily debilitating effects of cancer and its treatment that, over time, can wear them down. We therefore conclude that we would retain the complete SCH.

Automated self-management coaching alone had the least impact on lowering symptom burden. Historically, patient self-management instructions have been the first-line approach to symptom care. Self-management coaching through digital tools is common, although some systems providing self-management instruction require an additional, potentially burdensome step to access information, such as accessing a site or emailing instructions rather than providing active coaching during reporting as the SCH system does.^5,11,33,34,35^ Our study demonstrated that while self-management coaching alone did not provide optimal symptom control, it added value when combined with clinician follow-up for poorly controlled, higher-level symptoms.^5,8,10,13,34^

There were no significant differences in overall symptom reduction between self-management coaching groups, whether using a commercial activity tracker or not. Previous literature supports the use of activity trackers to obtain a comprehensive view of patient status^36^; limited studies have found that activity trackers are superior to ePRO alone.^37^ However, other studies have shown that activity tracker use improves physical activity,^38,39^ sleep,^40^ or fatigue.^41^ Further research is warranted to determine the value of adding peripheral devices to ePRO systems to improve symptom control.

The NP follow-up groups were significantly better at lowering symptom burden than self-management coaching alone. Clinician response is an important component to treat moderate-to-severe symptoms. Interestingly, individuals in the NP group who used the decision support system had outcomes similar to those in the NP group who used their best professional judgment. There has been limited study of decision support systems for symptom care or the degree to which clinicians incorporate widely available evidence-based symptom guidelines.^21,42^ To our knowledge, this is the first report of a randomized comparison of technology-aided clinician symptom care using automated decision support compared with NP professional judgment. Further study is warranted to explore clinician satisfaction and utilization of decision support systems and the degree to which professional judgment mirrors current guidelines.

Our SCH intervention used NPs to follow up on poorly controlled symptoms. Other ePRO systems have follow-up provided by registered nurses.^33,34,43^ We used NPs so that prescription changes could be streamlined. While we did not find improvement in symptom reduction with the decision support system compared with NP professional judgment alone, a decision support system might be beneficial for registered nurse decision support and could be tested in future studies.

While NP groups were superior to self-management coaching, the combined SCH intervention was more effective in lowering symptom burden to either self-management or clinician follow-up alone. This suggests potential synergy between patient self-management coaching and clinician follow-up. Digital tools can efficiently provide tailored, multicomponent approaches to improve symptom outcomes.^34,44,45^ While self-management coaching alone may not be sufficient to improve all symptoms, it may deescalate some, leaving fewer symptoms for clinicians to address and help patients keep symptom burden low over time once clinicians have intensified and tailored symptom care for the patient’s particular needs. The 2 tiers—automated self-coaching and, as-needed, NP follow-up—work in synergy and efficiently involve the oncology team only as warranted.

### Limitations

There are several limitations to our study. Of the 884 patients who consented, 109 (12.3%) never participated by providing a report to the SCH intervention. This is consistent with other behavioral studies in which patients express interest at enrollment but reconsider once home.^46,47^ In addition, 25.8% (n = 195) of 757 participants discontinued study participation before treatment protocol completion, although the mean (SD) study time was more than 2 months (72.6 [42.2] days). These issues of uptake and sustained participation are common with ePRO systems^48^ and warrant further study to determine best practices for engagement and sustainment.

## Conclusions

In this randomized clinical trial, ePRO symptom monitoring and management platforms that combined daily symptom assessment with automated self-management coaching and clinician follow-up for moderate-to-severe symptoms achieved the greatest symptom burden reduction for patients with cancer over the course of treatment. Cancer symptom burden may be substantially reduced through systematically applied digital solutions when both patient support and clinician notifications with follow-up are integrated.

## Notes

Supplement 1.
Trial Protocol and Statistical Analysis Plan

Supplement 2. **eFigure.** CONSORT Flow Diagram

Supplement 3.
Data Sharing Statement
