# Supplementary material for: Essential Components of an Electronic Patient-Reported Symptom Monitoring and Management System: A Randomized Clinical Trial
Source: JAMA Netw Open. 2024 Sep 13;7(9):e2433153. doi: 10.1001/jamanetworkopen.2024.33153 (PMC11400212; doi:10.1001/jamanetworkopen.2024.33153)
Supplement: Supplement 1. — Trial Protocol and Statistical Analysis Plan [file jamanetwopen-e2433153-s001.pdf]

Contents

Statistical Analysis 2
Study Protocol 4

Statistical Analysis

Statistical analyses were conducted in IBM SPSS Statistics for Windows, version 27 (IBM Corp., Armonk, N.Y., USA) and SAS/STAT software, Version 9.4 (SAS Institute Inc., Cary, NC, USA). Basic descriptive statistics were utilized for demographic and clinical metrics of patients separated by the five randomization groups. Proportion of call types are reported as are the means (SD) of total number of calls. Call compliance was based on the proportion of the number of calls with number of expected calls where expected calls were the number of days from when the participant was first entered into the call system till their last call subtracting any days where the participant was listed as being hospitalized and thus not expected to call. Symptom severity scores across all symptoms were the dependent variables while randomization group was the independent variable. Summary statistics for the 11 symptoms of interest included means and standard deviation (SD) for severity and the frequency and proportion of symptom reports utilizing a category based on the 0-10 severity scale including 0=symptom not endorsed, 1-3= mild, 4-7 moderate, and 8-10 severe. Means and SD are also reported for the number of calls endorsing at least one symptom in these severity categories. As previously done, we report on the proportion of patients that reported at least 1 moderate to severe symptom during the study as an indicator of the potential intervention utilization.[1]

Unconditional Inferential analysis. To facilitate visual comparisons, Figure 2 reflects the key assumption for randomized trials of equal population baseline means. For inference, the initial chance group randomization differences are adjusted formally through the analysis model. The constrained longitudinal data analysis model (cLDA) (Liang & Zeger, 2000; Lu, 2009) assumes a single baseline pre-randomization population, with differences emerging only after initiation of a randomized treatment. Following the pattern seen in the descriptive plots, and consistent with the Bayesian Information Criterion of alternative polynomial models, we fit a quadratic cLDA model, estimated under restricted maximum likelihood:

$$S_{itg} = \beta_{0i} + \beta_{1g} t + \beta_{2g} t^2 + \varepsilon_{igt}\ ,\ \ \varepsilon_{igt} \sim N(0, \sigma^2),\ \ \beta_{0i} \sim N(\beta, \psi)\text{, where}$$

S_{itg} is the Symptom Burden score for patient *i* at time *t* in treatment group *g*,

β_{0i} is the intercept for patient i, with normally distributed variance ψ and constant mean β_0 assumed equal across treatment arms at baseline,

β_{1g} and β_{2g} are the within-group regression coefficients for the linear and quadratic time trends, respectively,

and ε_{igt} is a normally distributed residual term. The provision of a random intercept (varying across individuals) allows for serial dependence in the longitudinal observations.

The likelihood estimates are valid under the assumption that missing longitudinal data for a treatment arm are in principle predictable from past observed data, even when the groups differ

[1] Mooney K, Beck SL, Friedman RH, Farzanfar, R, Wong B. (2014) Automated Monitoring of symptoms during ambulatory chemotherapy and oncology providers' use of the information: a randomized controlled clinical trial. Support Care Cancer. 22 (9):2343-2350.

in their proportions of missing data. All data points are incorporated in the analysis; in alignment with an intent-to-treat standard, no data points are imputed and none are deleted.

Since the model incorporates continuous time, summary quantitative effects and endpoints are estimated as the area under the population curves, representing the total amount of symptom burden experienced in population *g*. This endpoint defines an overall summary of how well the intervention worked, and we conduct all formal hypothesis tests using this endpoint. For each treatment arm, the total burden is computed as the integral of the quadratic curve. To simplify calculations, we standardize so that time runs from 0 to 1. Then the integral, or total area under the group curve up to time t is given by $AUC|t = (1/3)*b_2t^3 + (1/2)*b_1t^2 + b_0t$. Standardization simplifies the total AUC at t=1, the end of study, as AUC = (1/3)*b2 + (1/2)* b1 + b0. Net differences between pairs of treatment arms are obtained by subtracting the corresponding areas.

Conditional Inferential Analysis. The unconditional model permits tests of the primary intent-to-treat hypothesis of equal time trajectories across intervention groups. A secondary question concerns whether these relationships are consistent across a plausibly relevant set of pre-randomization covariates: cancer stage, cancer diagnosis, gender, age, working status, education, income, self-defined racial category, and study site (Utah or Georgia). To address this question, we follow a conventional regression strategy of retaining any significant covariate effects when added simultaneously to the unconditional model. Given randomization, covariate-by-intervention group interactions (that do not include time) are necessarily attributable to chance imbalance and are not considered in the conditional models.

Missing Data:

Missing data occurred on 1.4-2.1% of the daily symptom item scores. This small degree of missingness was handled by regression imputation for computation of the sum symptom burden scores if at least 7 item severity ratings (including an explicit 0 as a rating) were present for that day. If fewer than 7 individual symptom items were present for a day, the overall symptom burden score was coded as missing for that day.

Study Protocol

Symptom Care @ Home: Deconstructing an effective, technology-assisted, symptom management intervention.

Protocol Summary

Principal Investigator: Kathi Mooney, PhD, RN

University of Utah, College of Nursing

Huntsman Cancer Institute

kathi.mooney@nurs.utah.edu

Funded by: National Cancer Institute (R01CA206522)

Introduction and Rational

Cancer patients receiving chemotherapy experience multiple poorly controlled symptoms at home in the interim weeks between clinic visits and the next infusion[1,2]. Their care during this interim time period is suboptimal[2,3]. Technology can be utilized to bridge the gap between patient symptom needs at home and oncology team response[4,5]. Automated patient-reported symptom home monitoring and management systems offer a new approach to symptom care. Our research team has developed and tested SymptomCare@Home (SCH). This multi-component automated system monitors daily patient-reported symptoms at home, provides tailored automated self-management coaching based on the specific symptoms reported, and transmits unrelieved symptom alert reports to an oncology provider who, using the SCH decision support system, provides follow-up telephone-delivered symptom care. We have demonstrated that SCH dramatically reduces physical and psychological symptom severity. However there remain several gaps in translating a successful, multi-component symptom intervention into clinical practice. These include understanding 1) the contribution and value of each part of the intervention so that the active components are selected for clinical implementation and 2) the costs associated with the intervention and its components. Therefore, the aims of this project are to deconstruct the SCH symptom monitoring and management system to determine the relative contribution and costs of its components to achieving symptom reduction, maintain general health functioning and reduce healthcare utilization. We will also examine who benefits most and least from the individual components and from the overall system.

Methods

A prospective, longitudinal 5 group clinical trial with random assignment to group is planned for Aim 1 to deconstruct and identify the relative contribution of the intervention elements to achieving improved symptom control, overall health functioning and decreased health care utilization. Aim 2 also adds a historical usual care 6th group. The complete SCH system utilizes a telephone-based interactive voice response (IVR) automated system to collect patient-reported daily information about the presence and severity of 11 common symptoms of chemotherapy: fatigue, trouble sleeping, nausea and vomiting, pain, numbness or tingling, feeling blue or down, feeling nervous or anxious, distress over appearance, diarrhea, sore mouth, and trouble thinking or concentrating. It provides automated self-management coaching paired to these symptom reports. Alert reports about unrelieved symptoms are monitored by a study nurse practitioner who provides follow-up care using a symptom decision support guideline-based system. The 5 groups will include Group 1 (SCC) automated self-management coaching with activity tracker blinded; Group 2 (SCC +AT)Automated self-management coaching plus activity tracker with data visualization; Group 3 (NP) nurse practitioner follow-up calls when symptoms exceed alert thresholds; Group 4 (NP+ DSS) nurse practitioner follow-up calls utilizing the SCH decision support system when symptoms exceed alert thresholds; and Group 5 (SCH) the complete SCH intervention with all components used in the prior efficacy study (SCC+NP+DSS)

Inclusion/Exclusion Criteria

The study will use the following inclusion and exclusion criteria those: over 18 years, with a histological diagnosis of cancer, life expectancy of at least three months, beginning a new course of chemotherapy that is planned for a minimum of three cycles, English speaking, with daily access to a telephone and cognitively capable to use the phone unassisted as verified by study staff at recruitment. Patients are excluded if they are receiving concurrent radiation therapy, since they would already have daily contact with oncology care providers, or if they are receiving immunotherapy exclusively since key symptoms may not be covered in the chemotherapy focused 11 symptoms tracked. Patients will be compensated for their time receiving 2 payments of $75 each or an Activity Tracker at the end of the study if they prefer.

Randomization

The proposed study will use randomized experimental manipulations to determine and compare subcomponent impact on symptom burden. Participants will be randomly assigned to one of five treatment arms using REDCap. Patients beginning a course of cancer chemotherapy will participate in daily automated symptom reporting and be randomized to the following 4 treatment arms: **Group 1 (SCC)** automated self-management coaching blinded activity tracker; **Group 2** (SCC+AT) automated self-management coaching, activity tracker with data visualization; **Group 3 (NP)** nurse practitioner follow-up calls when symptoms exceed alert thresholds; **Group 4 (NP+ DSS)** nurse practitioner follow-up utilizing the SCH decision support system when symptoms exceed alert thresholds; and **Group 5 (SCH)** the complete SCH intervention with all three components including self-management coaching and nurse practitioner follow up utilizing the decision support system when symptoms exceed alert thresholds (SCC+NP+DSS).

Trial Conditions

We considered but chose not to include a usual care group. From our previous study we know the intervention is efficacious and significantly improves symptom experience, therefore, we do not have clinical equipoise for randomization to usual care as it would knowingly disadvantage participants. While we propose hypotheses for the study Aims, we believe that each group will benefit.

All groups receive one of the SCH components- the daily automated patient- reported symptom monitor of the 11 symptoms. We chose not to test this component for several reasons, first, the patient-reported data serves as one of the outcome measures and we wanted to keep the mode of symptom reporting the same since other studies have found that people are more likely to report higher symptom severity scores, especially for sensitive areas like depressed mood, when reported to an automated system as opposed to another person. Secondly, our previous research has shown that monitoring alone is not efficacious; it is a necessary but not an active ingredient of the intervention effect.

Automated algorithm-based self-management coaching (groups 1, 2, 5). Automated symptom self-management coaching is delivered during the monitoring call. Coaching is based on national evidence-based guidelines. Developed initially with a DOD grant and validated by a national panel of symptom experts, algorithms have been written that vary coaching content based on severity level (mild, moderate or severe), contextual responses (# of times vomited, low fluid volume, feeling dizzy) and whether this is a new symptom, has been reported previously and is getting better or reported previously and getting worse. Messages also have decision rules that include the number of times the identical message can be repeated in a specific period of time. Coaching is both for immediate action and other coaching is designed for behaviors that should be adopted long-term. Generally, these involve lifestyle change, for example, beginning an exercise program. Some messages are automatically played, and others ask the participant if they would like to hear the message, for example improving sleep. Coaching messages are reviewed and updated as new evidence and guideline revisions occur, we will review messages at the start of the study and then annually with content reprogrammed. On average the messages add about 30 seconds to call length. In our most recent study UC participant calls with no self-management coaching lasted 4 min19 sec and with self-management coaching calls were 4 min 45 sec.

Study nurse practitioner follow-up based on symptom alert reports (received by Groups 3, 4, 5) Participants are asked to call the SCH monitoring system in the morning so that the NP can provide follow-up calls in the afternoon including weekends and holidays. Alerts are automatically and immediately generated from the patient's daily call data based on preset thresholds. There are 29 different symptom responses that generate an alert for the 11 symptoms. Some alerts are generated for symptom severity at moderate or higher level (4-10 on 10-point scale) such as a pain severity rating of 6, while other thresholds require a certain pattern of response such as trouble sleeping at a moderately severe level (4-7 on a 1-10 scale) on 3 of the past 7 days. In addition, some alerts are generated for contextual responses such as an inability to ingest and retain 3 cups of fluid in the past 24 hours, or 5 or > episodes of vomiting in the past 24 hours. The NP logs into the SCH Alert and Call Data website which displays all of the patients who have called that day and met an alert threshold. Clicking on a patient's name brings up the call report showing what symptoms alerted and the severity levels and then below additional contextual responses to symptom 'drill down' questions (i.e., the number of times vomited in the past 24 hours). For Group 3, the NP considers the alert data

and then calls the patient to follow up and intensify care based on her/his best professional judgment.

Symptom clinical decision support system (DSS) (utilized in Groups 4, 5 receive NP follow-up care utilizing the DSS component. In addition to the SCH Alert and Call Data display, the DSS has several functions: (1) the DSS organizes and displays patient specific data, providing the symptom data the patient has provided over the course of the study in historical, cycle-based graphical trends (See Appendix B). The NP can view trends for a single symptom or overlay several symptoms. (2) It manages the workflow generated by the alert reports allowing orderly movement through the website first patient reported data, then a place to record additional assessment accessed from the EHR (for example a recent blood count or scan) and then assessments to ask the patient on the follow-up call. (3) There are also reminders that can be set for example to call the patient back and an area on the initial patient specific window that allows the NP to make a custom note (such as: patient prefers to be called Kathleen); it displays an alert-based symptom with the recommended follow-up assessment, pharmacological and non-pharmacological national guideline strategies as well as potential referrals. Check boxes are provided so the NP can indicate what she/he did; (4) it also provides pertinent patient specific health information, contact information, preferred pharmacy contact phone number, etc. and (5) it organizes a report of all relevant data assessed and actions taken from each phone encounter. The NP documents care in the DSS and can copy and paste it into the EHR. In addition, notes can be sent to the patient's oncologist, mid-level provider and/or nurse. The primary source for the study symptom intervention guidelines is the series of supportive care guidelines offered by the National Comprehensive Cancer Network (NCCN). Other sources utilized included the American Society of Clinical Oncology (ASCO), Multinational Association of Supportive Care in Cancer (MASCC) and Oncology Nursing Society Putting Evidence into Practice (PEP) and Cancer Care Ontario. These guidelines are based on research and best available evidence, developed and annually updated by expert panels, focused on clinical use with decision pathways outlining specific management and widely adopted within the oncology community. National guidelines will be updated at the start of the study and then reviewed annually with content reprogrammed as needed.

Commercial activity tracker (Group 1, 2). We will provide a Garmin activity tracker to evaluate acceptability and potential benefit in self-management for activity and sleep. eHealth tools are gaining widespread adoption among the public. With a focus for continuing to improve the automated SCH system, we see an opportunity to add and evaluate the inclusion of commercially available eHealth tools to augment the lifestyle self-management coaching for physical activity and monitoring sleep. The potential benefit of the activity tracker comes from the ability to self-monitor and gain immediate feedback on activity, exercise behavior and sleep. Activity trackers have been shown to promote increased physical activity even in sedentary people. In this study Group 1 will receive the activity tracker but not be able to see their data. Group 2 will be able to see and track their data. The activity tracker wristband captures and displays the time, daily steps total, distance travelled, calories, flights climbed, heart rate and sleep. For Group 2, data can be synced to a computer to provide an activity dashboard if the participant chooses. At study enrollment Group 1 and 2 participants will be instructed how to use the activity tracker for their group with step-by-step instructions will also be listed in their participant manual. Participants will be told to wear the activity tracker continuously during the study including at night, taking it off only to shower or for water sports. They will be assisted to set up their web account and provide permission for study access to the data as well. We will

not utilize the activity tracker data as outcome variables (physical activity or sleep). The purpose of providing the activity tracker is to extend the self- management intervention in Group 2 by comparing to Group 1 rather than outcome research data. Reliability of commercially available activity trackers is still being studied and established. We chose not to give Group 5 an activity tracker. Group 5 has all components of the SCH system that we tested in the efficacy study, and it serves as the comparison for the deconstructed elements from the efficacy study. Participants in Group 2 will have the activity tracker feedback plus the self-management coaching and will be compared with Group 1 who receives the self-management coaching component and wears the activity tracker that is blinded to the participant, allowing us to determine the value of adding an activity tracker to the self-management component.

With the 5-group design we will be able to determine the value to symptom control of: cognitive behavioral self-management coaching (Group 1); of an activity tracker paired with self-management coaching (Group 2); of nurse practitioner follow-up after symptom alerts (Group 3); and of automated decisional support for nurse practitioner use in follow-up after symptom alerts (Group 4). This will also allow us to understand how the self-management coaching, the NP and the DSS components each contributes to the benefit achieved from the complete, multi-component SCH intervention (Group 5) and the associated cost.

Treatment Integrity

Because components of the SCH system are automated, treatment fidelity is maintained because it is consistently delivered based on set algorithms. Thus, the collection of the patient reported data and the delivery of self-management coaching has no variation. However, NP adherence to delivery of the guideline-based follow-up care utilizing the DSS requires fidelity monitoring. We will document the care that is provided in Group 4 where the NPs provide care based on the DSS. In the previous study we developed an audit and feedback strategy where we systematically audited adherence to the DSS guidelines during the NP- Patient calls and with the documentation of care provided in the DSS system. For this study we will digitally record NP-patient telephone conversations. The procedures we use are based on recommended standards for fidelity audits. In the beginning we will audit all NPs calls in Groups 4 and provide feedback to the NP until compliance with the guidelines is consistently above 85% (80% is the standard for achieving high fidelity) then we will randomly audit 20% of calls and provide feedback to prevent intervention drift. Similarly, we will audit and document NP care in Group 3 by randomly auditing 20% of NP-patient calls but we will not provide feedback to the NPs.

Procedures

We will use the well-developed and successful procedures that we have developed over the course of our previous multi-site studies. The protocol manual from our recent efficacy trial will be adapted for this study. All staff will be trained in study procedures and will contribute to a daily log of any problems or exceptions to procedures that arise. Logs will be discussed with the Project Manager for resolution. Staff meetings will be held with the Project Manager, PI and Site investigators initially on a weekly basis and then as routines are stabilized move to bimonthly. Weekly audits will be run and circulated to the investigators and study staff providing summary data on the number of eligible participants screened, number of people approached, number of participants consented and retention rates by site and overall. In addition, we will conduct monthly audits to examine rates of missing data and to rectify any problems related to

missing data, so it is minimized. Issues from audits will be discussed at monthly research team conference call meetings and in other staff meetings. We have found that building a team culture where everyone shares in and is invested in tracking accrual, retention, data quality and completeness ensures best outcomes. We also set a climate for honesty and problem solving, encouraging disclosure without recrimination of protocol deviations or errors.

Screening, Recruitment, Enrollment and Retention of Participants

Potentially eligible participants will be identified from the infusion appointment rosters coordinated through the physician practices clinic schedule. Eligibility will be determined, and patients will be approached by trained study staff at the treatment planning visit and prior to their infusion. The study will be explained and informed consent obtained. Participants are then randomly assigned to one of the 5 treatment conditions using REDCap. Random assignment will be determined in blocks of 10 (at equal probability) with either two or three per arm per block (respectively), stratified by gender and independently at each of the 2 recruitment sites. We will recruit 878 participants, anticipating a 17% drop rate, to achieve 750 participants (150/group). Following randomization, staff will collect baseline data, orient the participant to the treatment arm assigned and train the participant in using the SCH system, providing them the study magnet and a participant study manual pertinent to their group assignment. SCH participant call-in adherence and study retention are keys to success and will be tracked closely. In our previous study, call adherence was high. SCH generates automated alert reports to study staff when a participant fails to call for several days. Study staff call these participants to trouble shoot issues and encourage participation. Retention is supported through these calls as well as study staff greeting participants on subsequent clinic and infusion visits. These methods have been successful in retaining participants; the dropout rate in our most recent study was 14% and we have planned a slightly more conservative 17% rate for this study.

Initial Power Calculations

For sample size estimation, we performed multilevel bootstrap resampling of the longitudinal sample available from the previous R01. Bootstrapping from this large sample is conservative, incorporating all error sources, both known and unknown. To specify effect magnitudes for the alternative hypotheses, we defined the previous two-arm mean effect difference as 100% and considered varied effect dispersal scenarios among the four experimental groups involved in hypotheses i and ii (including even dispersal: 0, 33, 67, 100%; maximal dispersal: 0, 0, 100, 100%; isolated effect: 0, 0, 0, 100%; and nuanced equipoised effect: 50, 50, 75, 100%). Based on repeated bootstrapped samples of 1000, group sample sizes of 145-160 reject the 6 df null hypothesis of equal mean conditional change at a rate greater than 85%.

Retaining approximately 150 participants per group will therefore conservatively yield power greater than 80%. 150 participants per group also yields standard errors for parameters and estimates that are reduced by more than a factor of 12 ($12=\sqrt{144}$), allowing for very precise estimation of individual values. Similar sample size requirements were obtained for activity tracking using data from another pilot study being conducted by Donaldson and colleagues. This pilot is demonstrating high acceptance and usability for the activity tracker technology. As an independent check, a two-tailed t-test conducted at $\alpha=.05/3$ (adjusting for the three outcomes) requires an average 150 per group for 80% power to detect small-to-moderate

effect sizes after conditioning on a predictive baseline, agreeing closely. We will recruit 175 participants/gp to retain 150/gp, allowing 17% attrition.

Proposed Analysis

Aim 1 Subcomponent impact on symptom burden. The proposed study will use randomized experimental manipulations to determine how it works. Participants will be randomly assigned to one of five treatment arms. Treatment arms 1-4 operationally define different levels of the SCH intervention implying a partial ordering of outcomes: (Hypothesis i) Arm 4 (NP + DSS) will prove superior to Arm 3 (NP), and (Hypothesis ii) Arm 5 (SCC+NP+DSS) will prove superior to Arm 4 (and therefore also to Arm 2). The hypotheses, as stated in the Aims, are logically equivalent (i.e., if and only if) to testing two independent hypotheses: (i) Arm 3 is superior to Arm 2, and (ii) Arm 4 is superior to Arm 3. Aim 1B introduces a new element to self-care coaching, activity tracking. This second arm (SCC-AT) will be compared directly with Arm 1 (SCC). Although the experimental design was guided by ordered scientific hypotheses, for conservatism all test statistics will two-sided.

Primary statistical hypotheses of effect. Two independent Aim 1A hypotheses i and ii will be evaluated under the comprehensive statistical hypothesis H_0: E(NP+DSS|Baseline) = E(NP|Baseline) *and* E(SCC+NP+DSS|Baseline) = E(NP+DSS|Baseline) versus H_1: *not* H_0, where *E* denotes Expectation. Aim 1B implies the single design hypothesis E(SCC-AT|Baseline) = E(SCC|Baseline). Conditioning on baseline adjusts for any imbalance following randomization that would otherwise introduce some degree of bias. The comprehensive hypothesis for both IA and IB will be evaluated jointly on three outcomes: overall health functioning, total symptom burden, and worst individual symptom. Crossing the design df with 3 df for the outcome measurements yields composite 6df and 3df tests for 1A and 1B, respectively, in a trivariate response mixed effects model. The overall composite hypothesis test controls the experiment-wise error rate to α=.05, with any follow-up comparisons requiring a significant overall global test.

The longitudinal model. We have considerable experience in implementing and analyzing intensive longitudinal data (Walls and Shafer, 2006) from daily or other frequent assessment schedules (107-110). For rating *m* on day *t* in randomized treatment group *g*, the main analytical model can be expressed as:

$y^{(g)}$

$$y^{(g)} = \Gamma X^{(g)} + a^{(g)} + b^{(g)}(t-1) + \varepsilon^{(g)},$$

$$a^{(g)}, b^{(g)} \sim \mathrm{N}([\alpha^{(g)}\ \beta^{(g)}]', \Psi),$$

$$\varepsilon^{(g)} \sim N(0, \sigma^2)$$

im(*t*>0)

*im*0

*i*0 *im* *im* *imt* *im* *im* *m* *m* *imt*

, where *a* and *b* denote individual therapeutic gain and rate-of-change, respectively; X is a vector of conventional fixed effect predictors; and y_{im0} is the baseline covariate that adjusts for randomization imbalance in clinical trials.

Treatment arm hypotheses are tested as customized contrasts on the mean intercept (α) and growth (β) parameter across treatment arms, as summarized above (e.g.,

$$H\ (i,ii):\ \sum \mathbf{K}\boldsymbol{\alpha}^{(g)} = \mathbf{0}$$

for Aim 1A, where K

encodes the six linear contrasts across mean treatment gain for both hypotheses i and ii jointly.

The generalized mixed effects approach not only controls experiment-wise error by testing the main hypotheses globally, but also documents the variances and covariances in the growth parameters for individuals. The random conditional intercept a_{im} represents gain or loss from baseline for each person, providing high- resolution estimation of individual change linked to treatment. Similarly, the slope coefficient b_{im} denotes the individual rate-of-change since the treatment began. Individual parameter variances scale generality and provide the first answer to Question 1, with follow-up analyses explaining this systematic change variation by expanding the set of predictors and moderators. One common modification allows a more flexible specification (such as lag one autoregression) for the repeated covariance matrix. The need for these modifications will be evaluated against the Bayesian Information Criterion. All analyses will be conducted as mixed effects models using SAS and their equivalent implementation in the multivariate setting in MPlus. Denominator degrees of freedom will be calculated using the conservative Kenward-Rogers method.

Aim 2 Cost-effectiveness analysis

We will conduct our cost-effectiveness analysis from two perspectives: the healthcare system and societal perspectives. The healthcare system perspective analysis will include direct medical costs such as those associated with outpatient, inpatient, and emergency room visits, as well as the opportunity cost of provider time. The societal perspective analysis will combine the healthcare system costs along with costs from the patient perspective such as direct non-medical costs (e.g., transportation costs for visits) and indirect or productivity costs (e.g., the opportunity costs for the time required to seek medical care).

<u>Study Design</u>: We will conduct this cost-effectiveness analysis using a decision analytic computer simulation model. This model will include 6 groups: the 5 groups described in Aim 1 along with usual care (Group 6- historical data from our last RCT N=178). As summarized in Table 2, our model will consist of 5 parameters: (1) intervention costs, (2) healthcare utilization, (3) direct medical costs, (4) productivity costs, and (5) quality- adjusted life-years (QALYs), which will be our measure of effectiveness. The values for these parameters will be derived from a combination of our previous work, the analyses undertaken through Aim 1, and the published literature.

References

1. Reilly CM, Bruner DW, Mitchell SA, et al. A literature synthesis of symptom prevalence and severity in persons receiving active cancer treatment. *Support Care Cancer*. 2013;21(6):1525-1550. doi:10.1007/s00520-012-1688-0

2. Cleeland CS, Zhao F, Chang VT, et al. The symptom burden of cancer: Evidence for a core set of cancer-related and treatment-related symptoms from the Eastern Cooperative Oncology Group Symptom Outcomes and Practice Patterns study. *Cancer*. 2013;119(24):4333-4340. doi:10.1002/cncr.28376

3. Mooney KH, Beck SL, Friedman RH, Farzanfar R, Wong B. Automated monitoring of symptoms during ambulatory chemotherapy and oncology providers' use of the information: a randomized controlled clinical trial. *Support Care Cancer*. 2014;22(9):2343-2350. doi:10.1007/s00520-014-2216-1

4. Mooney KH, Beck SL, Wong B, et al. Automated home monitoring and management of patient-reported symptoms during chemotherapy: results of the symptom care at home RCT. *Cancer Med*. 2017;6(3):537-546. doi:10.1002/cam4.1002

5. Berry DL, Hong F, Halpenny B, et al. Electronic Self-Report Assessment for Cancer and Self-Care Support: Results of a Multicenter Randomized Trial. *J Clin Oncol*. 2014;32(3):199-205. doi:10.1200/JCO.2013.48.6662
