# Supplementary material for: Essential Components of an Electronic Patient-Reported Symptom Monitoring and Management System: A Randomized Clinical Trial
Source: JAMA Netw Open. 2024 Sep 13;7(9):e2433153. doi: 10.1001/jamanetworkopen.2024.33153 (PMC11400212; doi:10.1001/jamanetworkopen.2024.33153)
Supplement: Supplement 2. — eFigure. CONSORT Flow Diagram [file jamanetwopen-e2433153-s002.pdf]

Supplemental Online Content

Mooney K, Gullatte M, Iacob E, et al. Essential components of an electronic patient-reported symptom monitoring and management system: a randomized clinical trial. *JAMA Netw Open*. 2024;7(9):e2433153. doi:10.1001/jamanetworkopen.2024.33153

eFigure. CONSORT Flow Diagram

This supplemental material has been provided by the authors to give readers additional information about their work.

© 2024 Mooney K et al. *JAMA Network Open*.

eFigure. CONSORT Flow Diagram

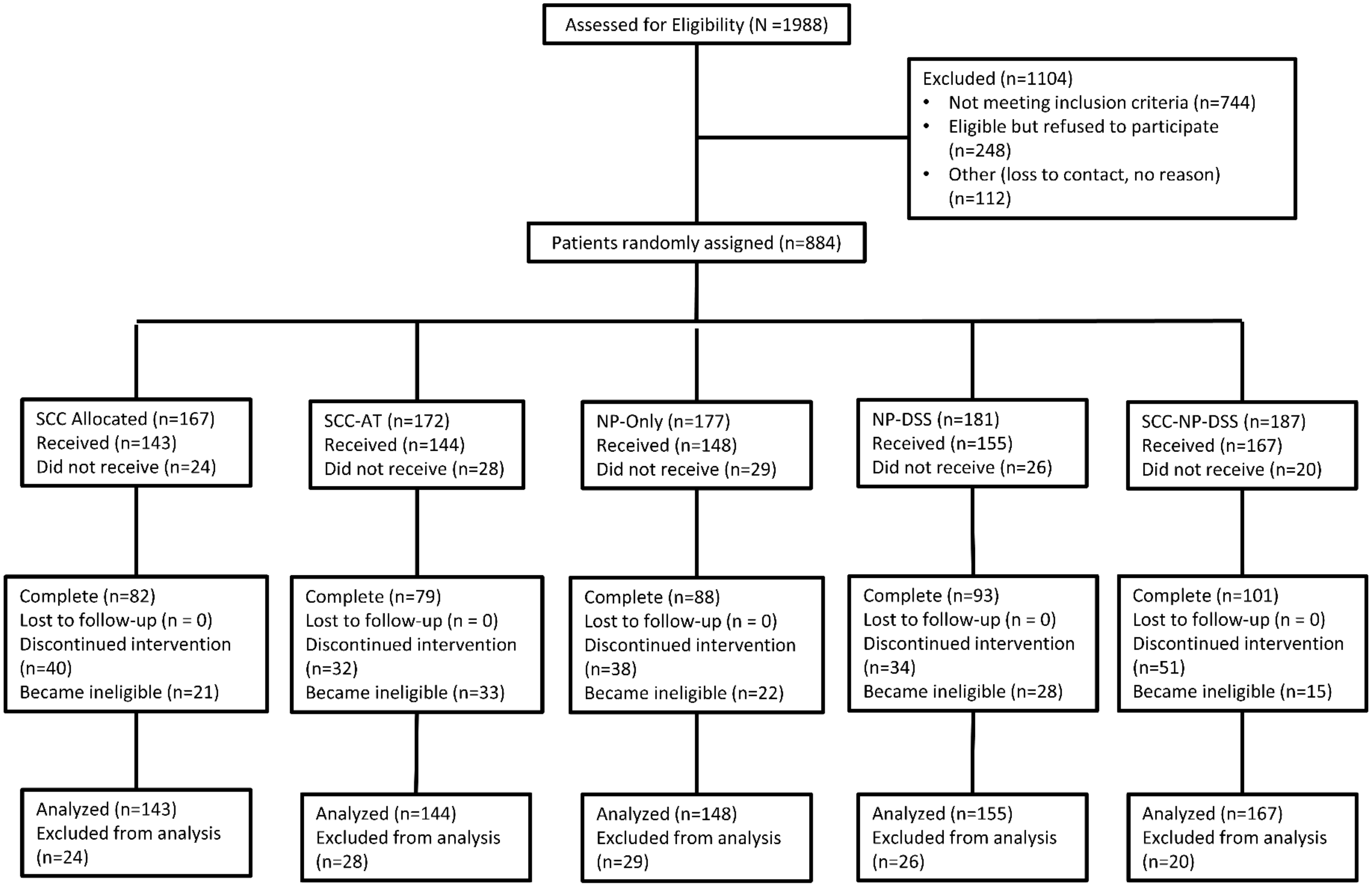

© 2024 Mooney K et al. *JAMA Network Open*.
