# Supplementary material for: Essential Components of an Electronic Patient-Reported Symptom Monitoring and Management System: A Randomized Clinical Trial
Source: JAMA Netw Open. 2024 Sep 13;7(9):e2433153. doi: 10.1001/jamanetworkopen.2024.33153 (PMC11400212; doi:10.1001/jamanetworkopen.2024.33153)
Supplement: Supplement 3. — Data Sharing Statement [file jamanetwopen-e2433153-s003.pdf]

Data Sharing Statement

Mooney. Essential Components of an Electronic Patient-Reported Symptom Monitoring and Management System. *JAMA Netw Open*. Published September 13, 2024.
doi:10.1001/jamanetworkopen.2024.33153

Data
Data available: Yes
Data types: Deidentified participant data
How to access data: Data are available from the corresponding author (kathi.mooney@nurs.utah.edu) upon reasonable request.
When available: beginning date: 05-31-2026

Supporting Documents
Document types: None

Additional Information
Who can access the data: Researchers whose proposed use of the data has been approved.
Types of analyses: For specified purpose that has been approved.
Mechanisms of data availability: After approval of a proposal and with signed data access agreement.
